# Oral Microbiome Link to Neurodegeneration in Glaucoma

**DOI:** 10.1371/journal.pone.0104416

**Published:** 2014-09-02

**Authors:** Konstantin Astafurov, Eman Elhawy, Lizhen Ren, Cecilia Q. Dong, Christina Igboin, Leslie Hyman, Ann Griffen, Thomas Mittag, John Danias

**Affiliations:** 1 Department of Cell Biology, State University of New York (SUNY) Downstate Medical Center, Brooklyn, New York, United States of America; 2 Department of Ophthalmology, State University of New York (SUNY) Downstate Medical Center, Brooklyn, New York, United States of America; 3 Division of Pediatric Dentistry, Ohio State University, Columbus, Ohio, United States of America; 4 Department of Preventive Medicine, Stony Brook University Medical Center, Stony Brook, New York, United States of America; 5 State University of New York (SUNY) Eye Institute, Brooklyn, New York, United States of America; 6 Department of Ophthalmology, Mount Sinai School of Medicine, New York, New York, United States of America; Dalhousie University, Canada

## Abstract

**Background:**

Glaucoma is a progressive optic nerve degenerative disease that often leads to blindness. Local inflammatory responses are implicated in the pathology of glaucoma. Although inflammatory episodes outside the CNS, such as those due to acute systemic infections, have been linked to central neurodegeneration, they do not appear to be relevant to glaucoma. Based on clinical observations, we hypothesized that chronic subclinical peripheral inflammation contributes to neurodegeneration in glaucoma.

**Methods:**

Mouthwash specimens from patients with glaucoma and control subjects were analyzed for the amount of bacteria. To determine a possible pathogenic mechanism, low-dose subcutaneous lipopolysaccharide (LPS) was administered in two separate animal models of glaucoma. Glaucomatous neurodegeneration was assessed in the retina and optic nerve two months later. Changes in gene expression of toll-like receptor 4 (TLR4) signaling pathway and complement as well as changes in microglial numbers and morphology were analyzed in the retina and optic nerve. The effect of pharmacologic blockade of TLR4 with naloxone was determined.

**Findings:**

Patients with glaucoma had higher bacterial oral counts compared to control subjects (p<0.017). Low-dose LPS administration in glaucoma animal models resulted in enhancement of axonal degeneration and neuronal loss. Microglial activation in the optic nerve and retina as well as upregulation of TLR4 signaling and complement system were observed. Pharmacologic blockade of TLR4 partially ameliorated the enhanced damage.

**Conclusions:**

The above findings suggest that the oral microbiome contributes to glaucoma pathophysiology. A plausible mechanism by which increased bacterial loads can lead to neurodegeneration is provided by experiments in animal models of the disease and involves activation of microglia in the retina and optic nerve, mediated through TLR4 signaling and complement upregulation. The finding that commensal bacteria may play a role in the development and/or progression of glaucomatous pathology may also be relevant to other chronic neurodegenerative disorders.

## Introduction

Glaucoma is a chronic neurodegenerative disease that affects the retinal ganglion cells (RGCs) in the neural retina and their axons in the optic nerve. It is the second most common cause of blindness worldwide [1]. Elevated intraocular pressure (IOP) is the most significant risk factor for the development and progression of the disease; however, its contribution is modest [2]. Similar to other neurodegenerative diseases, the contribution of known genetic factors in glaucoma is limited, with genes identified to date accounting for only 3–5% of cases of glaucoma in unselected populations [3].

Local inflammatory responses occurring at the site of pathology have been linked to neurodegeneration in glaucoma [4]–[6] as well as in other central nervous system (CNS) disorders such as Alzheimer's disease (AD) and Parkinson's disease (PD) [7], [8]. However, whether peripheral inflammation (that occurring outside the CNS) affects this process is less clear. Recently it has been shown that the risk of developing AD significantly increases following a peripheral infection [9] and that systemic infections can lead to faster cognitive decline [10]. A similar finding has been reported in an animal model of PD where peripheral over-expression of IL-1β accelerated the disease process [11]. These studies suggest that overt peripheral inflammation can exacerbate neurodegeneration. However, such inflammatory episodes cannot explain the bulk of neurodegeneration as most patients do not develop overt inflammation or have few such episodes during the course of their disease. Similar to other neurodegenerative disease acute inflammatory episodes cannot explain the majority of progression in cases of glaucoma. Clinical observations in glaucoma patients (JD), led us to hypothesize that *chronic subclinical* inflammatory processes such as that caused by microbiota colonizing humans may exacerbate neurodegeneration in glaucoma. We tested this hypothesis by analyzing human samples and utilizing animal models of glaucoma.

We determined the oral bacterial loads of patients with glaucoma (cases) and controls. Cases had significantly higher oral bacterial loads than controls without the disease. To confirm that glaucomatous neurodegeneration is affected by peripheral inflammation, we initially studied the effect of peripherally administered bacterial lipopolysaccharide (LPS) on glaucoma pathology. We used low doses of subcutaneous LPS to allow for prolonged exposure [12] and better reproduce the conditions expected to be elicited by chronic subclinical local bacterial infections. We utilized two separate mouse models of glaucoma – a spontaneous model (the DBA/2J) and an induced one (the microbead-induced model in C57BL/6 mice). Peripheral administration of LPS led to enhancement of glaucomatous damage in the retina and optic nerve two months later in both models. We then sought to identify the molecular and cellular mechanisms that would be responsible for such damage acceleration and discovered that LPS treatment induced significant TLR4 and complement system upregulation in the retinas of animals exposed to LPS compared to controls. In addition, pharmacologic blockage of TLR4 pathway with a TLR4 inhibitor (naloxone) partially prevented the damage in both retina and the optic nerve.

Identifying oral microbiome and peripheral inflammation as potential contributing factors to neurodegeneration in glaucoma may ultimately lead to discovery of novel drug targets and therapeutic interventions to control or limit this process.

## Results

**Figure 1:**
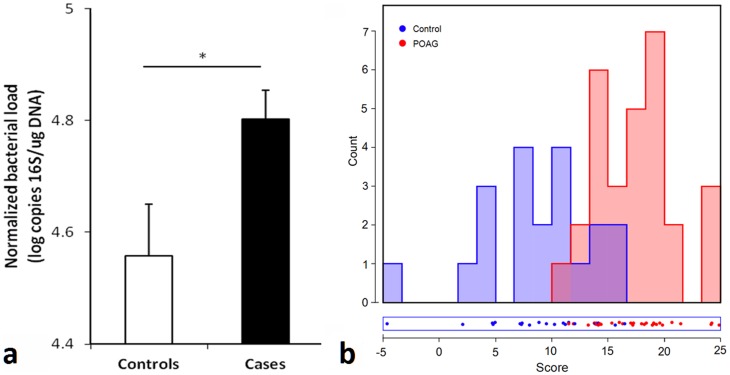
Difference in oral bacterial load in African-American patients with and without glaucoma. **a)** Normalized oral bacterial load (NOBL) of patients with and without glaucoma (p<0.017, t-test). Although cases were significantly different from controls in age (p<0.008, t-test), gender (p<0.02 Chi-square) and diabetes status (p<0.021, Chi square), GLM ANOVA of NOBLs (using group, gender, diabetes status and age [above or below median value] as independent variables) revealed a significant effect of group status (whether a subject belonged to cases or controls) only (p<0.024) while all other parameters did not show a statistically significant effect (p>0.26 for all). Linear regression of NOBL with age revealed a significant but low correlation (p<0.011, R^2^ = 0.063).**b)** Linear discriminant analysis of DNA amounts from various bacterial families normalized by the amount of total DNA in each sample. Cases are different from controls (MANOVA, p<0.001).

To investigate whether bacteria colonizing the oral cavity are associated with glaucomatous pathology we determined whether bacterial loads from mouthwash specimens in patients of the same racial group presenting at the SUNY Downstate Ophthalmology Clinic differed between cases (patients with glaucoma) and controls. Criteria for inclusion of glaucoma cases were open angles, typical glaucomatous visual field loss and optic nerve head (ONH) cupping (cup to disc ratio (CDR)>0.8). Control patients had normal IOP levels, healthy ONHs with CDR<0.5 and no significant ONH asymmetry. Quantitative assessment of the amount of 16S rRNA in 103 mouthwash specimens (58 cases and 45 controls) revealed a significant difference with cases having higher bacterial loads (**Figure 1a**, p<0.017, t-test). Subjects with bacterial loads in the higher quartile had over three times higher chance of having glaucoma (OR = 3.25 CI 1.20–10.55, p<0.015). Pyrosequencing of bacterial species in a subset of samples (27 cases and 20 controls) revealed that both Gram-positive and Gram–negative bacterial loads were significantly higher among cases than in controls (p<0.004 and p<0.016 respectively, t-test). Linear discriminant analysis confirmed that cases and controls were significantly different (eigenvalue 0.384, canonical correlation coefficient 0.526, Wilks lambda 0.722,) (**Figure 1b**, MANOVA, p<0.001).

**Figure 2:**
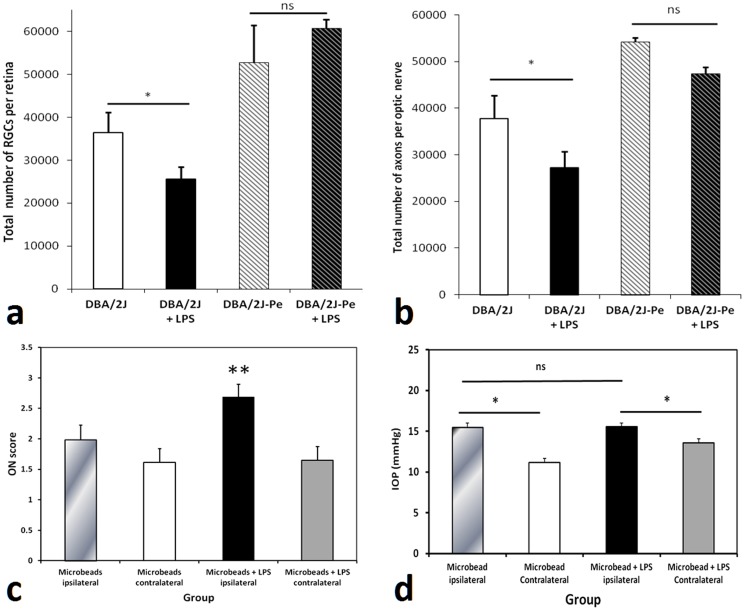
Peripheral LPS administration significantly accelerates glaucomatous pathology in DBA/2J mice (but not in DBA/2J-Pe mice) as well as in the microbead-induced IOP elevation model of glaucoma in C57BL/6 mice. LPS (60 µg) was injected into one hind footpad of 6-month old male DBA/2J and DBA/2J-Pe mice and retinal and optic nerve damage was assessed at 8 months of age. (**a**) Computer-assisted counts of total RGCs per retina (n = 18 and n = 24 retinas for DBA/2J and DBA/2J+LPS, respectively; n = 4 and n = 10 retinas for DBA/2J-Pe and DBA/2J-Pe+LPS, respectively). (**b**) Semi-automated total optic nerve axon counts, n = 9 and n = 19 optic nerves for DBA/2J and DBA/2J+LPS, respectively; n = 4 and n = 11 optic nerves for DBA/2J-Pe and DBA/2J-Pe+LPS respectively. The same amount of LPS (60 µg) or vehicle was also administered to male C57BL/6 mice that were (n = 16) or were not (n = 13) subjected to unilateral microbead-induced IOP elevation. optic nerve damage was assessed 2 months later. (**c**) Semi-quantitative optic nerve damage score in eyes of microbead-treated C57BL/6 animals. (**d**) Average IOP of eyes of microbead-treated C57BL/6 animals. Data are presented as mean ± SEM. Statistical differences were assessed by one-way ANOVA, followed by Tukey-Kramer post-hoc testing (*p<0.05, **p<0.01, ns>0.05).

To determine a possible pathogenic mechanism behind our finding in humans we then performed peripheral LPS-administration experiments in DBA/2J mice. This strain exhibits typical features of late-onset IOP-dependent optic nerve axon degeneration and RGC loss [13]–[16]. Optic nerve axon loss is apparent starting at ∼9 months of age and RGC loss follows. Female mice develop damage earlier than males with the latter having only minor optic nerve axon damage and RGC loss at 8 months of age [15]. A single subcutaneous administration of 60 µg of LPS into the footpad of male DBA/2J mice at 6 months of age led to a significant increase of optic nerve axon and RGC loss at 8 months of age by both direct measurement of RGCs and axons (**Figure 2a,b**) and semi-quantitative assessment (**Figure S1 a,b in File S1**). Because of the good correlation between the two methods (**Figure S1 c,d in File S1**), we utilized semi-quantitative assessment of RGCs and optic nerve axon counts for many of the experiments described below.

IOP change from baseline was not significantly different between groups (p>0.1, RM-ANOVA), thus excluding higher IOP as the cause for increased axonal and RGC degeneration. To exclude the possibility of cellular infiltration of the retina and optic nerve (as seen after intraperitoneal administration of high doses of LPS in susceptible mouse strains [17]) we sacrificed mice 2 weeks after peripheral LPS administration and examined the eyes histologically. No inflammatory cell infiltration was identified in either the retina or optic nerve.

To establish the specificity of the LPS response to glaucoma and the absence of a genetic background effect, we used 2 additional mouse strains as controls: DBA/2J-Pe mice (JAX: DBA/2J-Ap3b1^pe-8J^/J, Stock 002510) and C57BL/6 mice. DBA/2J-Pe animals are on the DBA/2 background and carry the *Ape3b-1*gene mutation that affects melanosomal maturation and thus renders them resistant to IOP elevation. DBA/2J-Pe do not develop axonal and RGC neurodegeneration [16]. LPS administered to 6 month old male DBA/2J-Pe mice and also to C57BL/6 mice caused no damage in the retina or optic nerve at 8 months of age when compared to untreated animals (**Figure 2a,b** and **Figure S1a,b in File S1**) (data not shown for C57BL/6).

We also investigated the effects of peripheral LPS administration in a separate animal glaucoma model utilizing microbead injection in C57BL/6 mice [18]. This model involves mechanical occlusion of the aqueous humor outflow pathway, which leads to IOP elevation and subsequent RGC and optic nerve axon degeneration two months post-treatment. Eyes with microbead-induced IOP elevation in mice subjected to peripheral subcutaneous LPS administration as above had significantly more optic nerve axon loss than in animals not exposed to LPS (**Figure 2c**). The mean IOP in microbead–injected eyes of LPS treated animals was not significantly different from that in microbead-injected eyes of non-LPS treated animals, thus eliminating higher IOP as the cause of the LPS effect (RM-ANOVA, post-hoc comparison p>0.05) (**Figure 2d**).

After LPS administration, mice of all backgrounds typically exhibit transient weight loss. We determined whether this response correlates with eye pathology in a group of DBA/2J mice. The average number of surviving RGCs in the retinas of each animal and the number of days it took animals to regain baseline weight correlated strongly (R^2^ = 0.98) (**Figure S2 in File S1**). This result suggests that the degree of response to LPS can modulate glaucomatous pathology in this animal model and, by extension, that other peripheral inflammatory episodes can potentially increase glaucomatous neurodegeneration.

We then attempted to determine a plausible mechanism by which higher oral bacterial loads and by extension peripheral inflammation may affect glaucomatous neurodegeneration.

**Figure 3:**
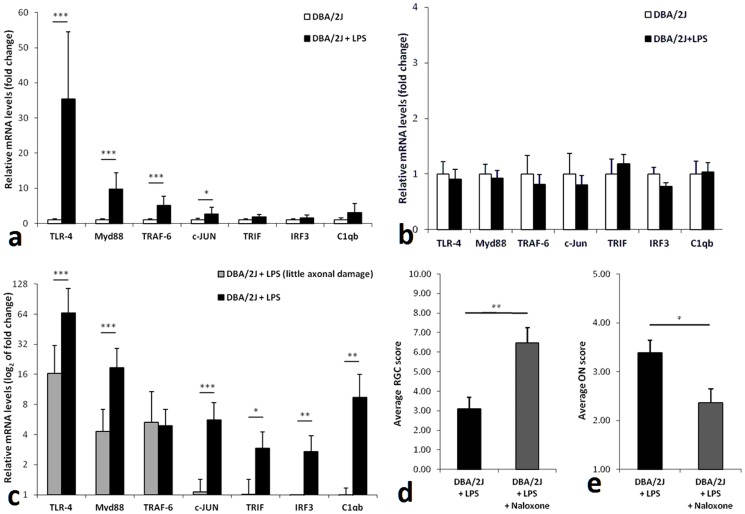
LPS induces up-regulation of TLR4 pathway and complement system genes in the retina but not in the brain of DBA/2J mice. Treatment with naloxone ameliorates both optic nerve axon and RGC loss. (**a**) qPCR analysis of relative mRNA levels of genes in the TLR pathways in retinas (n = 9) of LPS-treated mice compared to those of control mice (n = 9) (mRNA levels in control retinas were set at one). (**b**) Analysis of relative gene expression of the same genes in the brains of the same control and experimental mice. (**c**) Analysis of TLR and complement gene expression in retinas of LPS-treated mice segregated into two groups according to the extent of damage in the optic nerve: samples from “DBA/2J+LPS” group (n = 5) had significant axon loss, whereas samples from “DBA/2J+LPS (little axonal damage)” (n = 4) had only a small degree of axon loss (comparable to that of non-LPS treated male DBA2/J animals). (**d**) Analysis of RGC survival in eyes from LPS and naloxone (n = 16) and LPS only treated (n = 21) animals, using semi-quantitative scoring. (**e**) optic nerve scores of eyes from LPS and naloxone (n = 14) and LPS only (n = 15) treated animals using semi-quantitative assessment. Data were tested for statistical differences between groups using either one-way ANOVA followed by Tukey-Kramer post-hoc testing (multiple groups) or t-test (two groups) (*p<0.05, **p<0.01, ***p<0.001). All data are presented as mean ± SEM.

Toll-like receptors (TLR) are potential participants of such immune-mediated effects. TLR pathways have been implicated in the pathology of various neurodegenerations [19] as well as in glaucoma [20], [21]. We determined that LPS administration in DBA/2J mice led to significant upregulation of the Myd88-dependent pathway genes (Myd88, Traf6, cJun) which are downstream targets of TLR4 (the main receptor for LPS) in retinas of LPS treated mice (**Figure 3a**). Changes in the Myd88-independent downstream pathway genes Trif and Irf3 did not reach statistical significance (**Figure 3a**). Importantly, up-regulation of the same TLR4-pathway genes was not detected in the brains of the same animals (**Figure 3b**) indicating that this upregulation is specific for the retina in glaucoma.

To determine whether TLR pathway upregulation is relevant to the development of optic nerve axon loss we compared TLR pathway gene expression levels with the level of damage in individual eyes. Eyes with significantly more severe optic nerve axon damage had a higher-fold increase in relative mRNA expression for most of the TLR pathway genes tested.(**Figure 3c**). Additionally, complement component C1q (**Figure 3c**), was significantly more up-regulated in eyes with greater optic nerve axon damage. As shown recently, C1q absence results in amelioration of the optic nerve axon and RGC loss in the DBA/2 animal model [22]. The level of optic nerve axon loss correlated strongly with the level of upregulation of individual genes (**Figure S3 in File S1**).

We next sought to determine whether inhibition of TLR4 pathway could protect from glaucomatous damage. Naloxone is a partial TLR4 inhibitor [23] and has been shown to decrease microglial activation [24], while not affecting IOP [25]. Chronic administration of naloxone *in-vivo* via osmotic minipumps led to significant protection of RGCs (p<0.0024) (**Figure 3d**) and optic nerve axons (p<0.014) (**Figure 3e**) in DBA2/J animals.

**Figure 4:**
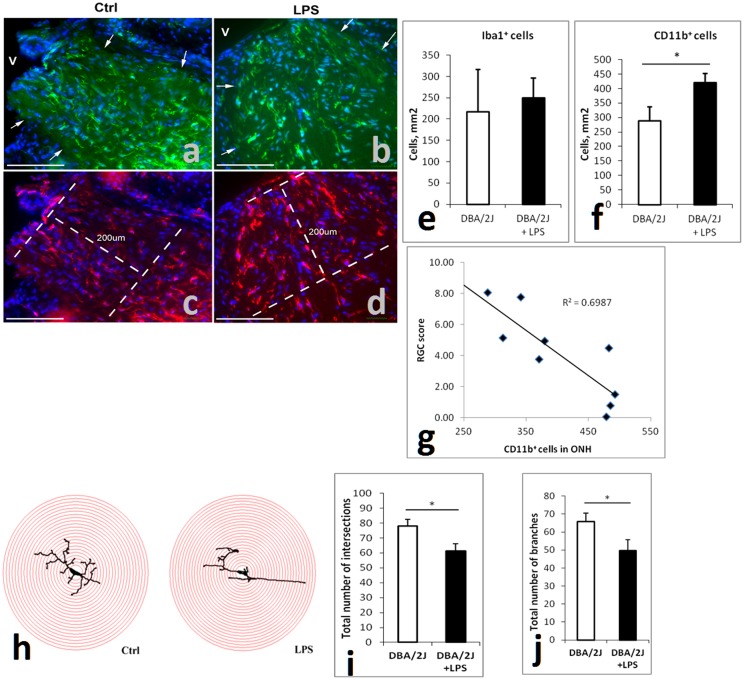
Peripheral LPS administration leads to microglial cell changes in the ONH and retina. (**a–d**) Representative images of Iba1 (green)/DAPI (blue) (a,b), as well as CD11b (red)/DAPI(blue) (c,d)-stained optic nerve head tissue from DBA2/J animals treated without (a,c) or with LPS (b,d). “V” denotes location of the vitreous body. Arrows point to the junction between the optic nerve tissue and the sclera. Dashed lines outline the region where cell counts were performed. Scale bars are 100 µm. (**e,f**) Analysis of Iba1^+^ (e) and CD11b^+^ (f) microglial cell numbers in the proximal unmyelinated part of the ONH. Counts are average numbers from 6 and 7 LPS treated eyes and 3 and 4 controls respectively. (**g**) Correlation between RGC score and numbers of CD11b^+^ cells in the prelaminar region of the ONH of all eyes analyzed. (**h**) Sholl analysis of retinal microglial cells: representative images of microglial cell traces subjected to Sholl analysis with the overlaid concentric circles. (**i**) The total number of intersections was decreased in microglia from the retinas of LPS-treated animals (n = 8) as compared to controls (n = 6). (**j**) Skeleton analysis of retinal microglial cell: the retinal microglia of LPS-treated mice (n = 8 eyes) had fewer branches than that of control mice (n = 6 eyes). Assessments of statistically significant differences between groups were done using t-test (*p<0.05).

Microglia are involved in glaucomatous [6] and other neurodegenerative disease [19] pathology and express high levels of TLR4. Although numbers of Iba1^+^ cells (a pan-microglial marker) were not different, CD11b^+^ cells (indicative of activated microglia [26]) were more abundant in the optic nerve pre-laminar region of LPS-treated compared to control mice (**Figure 4a–f**) and their number correlated with RGC loss (**Figure 4g**) suggesting that peripheral LPS administration leads to local activation of microglia in the ONH. This may potentially occur due to the inherent “leakiness” of capillaries in this region [27], IOP-induced changes in capillary permeability or infiltration by peripheral monocytes as has been suggested [28].

The total number of Iba1^+^, amoeboid Iba1^+^, Iba1^+^/CD68^+^, or CD68^+^-only cells in the retinal nerve fiber layer (NFL) and RGC-layer (**Figure S4 in File S1**) was not different between groups.

To detect subtle changes in microglial activation, we performed Sholl (**Figure 4h,i**), skeleton (**Figure 4j**) and morphological (**Figure S5 in File S1**) analysis. Both Sholl and skeleton analysis have been used to examine changes in microglial dynamic behavior and morphology [29]. Retinal microglia from LPS treated mice had decreased total number of intersections and branches compared to control mice (**Figure 4i,j**) as well as other parameters (**Figure S5 in File S1**) indicating a transition to a more activated state.

Despite the apparent microglial activation, qPCR of known markers of such activation did not show significant expression/upregulation after LPS-treatment in the retina (**Figure S6 in File S1**).

## Discussion

Acute peripheral inflammation, caused by infection, or artificially induced via an injection of various inflammogens (e.g. LPS), is known to produce rapid changes in the functioning of the CNS collectively known as “sickness behavior” and that may include loss of appetite, increase in fatigue, changes in sleep patterns, social aversion, deficits in cognitive and motor function [30]. Some studies have suggested that acute infections and inflammation in the periphery may even be associated with central neurodegeneration and may confer a greater risk of developing or accelerating neurodegenerative diseases such as AD [9]. However, *clinically-evident* inflammatory episodes are not known to be more frequent in patients with glaucoma and other neurodegenerative disease as compared to unaffected individuals; thus, acute inflammatory events cannot account for the bulk of neurodegeneration. By contrast, chronic sub-clinical inflammation initiated by microbiota that colonize humans is not uncommon and has been linked to a number of human pathologies and conditions such as atherosclerosis, peripheral artery disease [31] and obesity [32]. In addition, recent studies have demonstrated that chronic, subacute inflammation can affect CNS homeostasis as well. For example, it was shown that induction of a very low-grade endotoxemia can impair declarative memory [33], whereas epidemiological investigations have revealed an association between low-grade peripheral inflammation and age-related decline in cognitive function [34],[35]. On the basis of this evidence and clinical observations (JD) we hypothesized that such chronic sub-clinical inflammation may have a role in exacerbating or augmenting neurodegeneration in glaucoma.

Several previous studies have examined whether infection with *Helicobacter pylori*, a bacterium responsible for chronic inflammation of the gastric mucosa, is implicated in glaucoma pathology. While some studies have suggested an association between the presence of *H. pylori* and glaucoma [36], [37], other studies have failed to confirm these findings [38]. We hypothesized that the overall bacterial load rather than presence of an individual species would be more relevant for inducing inflammatory responses affecting glaucoma pathology. Based on the reported link between oral microbiota and human pathology [31], we further explored the link between oral microbiota and glaucoma. We utilized total oral bacterial load in mouthwash specimens as a surrogate of the bacterial load present in the periodontal space. In a cross-sectional pilot human study we showed that patients with glaucoma had higher oral bacterial loads. Subjects with oral bacteria loads in the upper quartile were over three times more likely to have the disease. Although this cross-sectional study has certain limitations and does not prove causation, it suggests a link between glaucomatous neurodegeneration and oral health.

To understand how oral bacteria may mediate such an effect, we studied animal models of the disease. We used low levels of LPS administered peripherally subcutaneously to simulate chronic subclinical peripheral inflammation. In two separate animal models of glaucoma such exposure to LPS results in enhanced neurodegeneration. This effect appears to be mediated through upregulation of the TLR4 and the complement system pathways which result in microglial activation in the pathological tissue, but not other CNS tissue. Interestingly, recent studies in humans have also identified an association between TLR4 polymorphisms and normal tension glaucoma [39]. Our experimental animal data suggest that the elevated IOP causes specific targeting of the optic nerve head (and probably later the retina) to peripheral bacterially-induced inflammation in glaucoma (presumably the same applies to humans with glaucoma). This unique “susceptibility” of these CNS tissues in glaucoma appears to be IOP-dependent as control animals treated with LPS alone do not develop significant neurodegeneration.

Previous studies in DBA/2J mice have suggested that migration of circulating monocytes into the optic nerve head was a critical step in the initiation of damage in this animal model [28]. In the current work, we demonstrated an increase in the number of Cd11b+ cells in the prelaminar region of the ONH following LPS administration, but we didn't determine the origin of these cells. It is thus unclear whether these represented migrating monocytes or rather activated local resident microglial cells. Future experiments will address this important question.

In addition to detecting changes in microglial numbers in the ONH we detected microglial activation within the RGC and NFL layers by morphometric criteria. At the same time, we did not observe statistically significant changes in common markers of microglial activation. Anecdotally, we did not observe significant morphologic changes in microglial cells in deeper retinal layers between LPS-treated mice and controls. This can be accounted for by the fact that the whole retinas were utilized for qPCR experiments. Hence, the sensitivity of the qPCR assays might have not been sufficient to detect changes in the markers of activation of this subset of microglial cells. An alternative explanation is that microglial cells alter their expression of common activation markers early upon activation and then revert to baseline while maintaining their morphological changes for longer periods of time. Thus, a more careful investigation of the dynamics of microglial activation in the retina (looking at multiple time points as well as at various microglial subsets) will provide a clearer picture of microglial cells contribution to glaucomatous damage in this model.

Since the level of TLR4 upregulation was associated with the amount of neurodegeneration in this animal model, we used naloxone to pharmacologically inhibit this pathway and investigate its contribution to this process. Naloxone is a competitive inhibitor of TLR4 signaling [40]. Besides its recently discovered TLR4 antagonism, naloxone is primarily known for its antagonism of the opioid receptors [41]. The “neuroprotection” afforded by naloxone in this case is unlikely to be the result of its action on opioid receptors as opioid agonists (such as morphine) have been shown to be protective in various models of retinal neurodegeneration (including a rabbit retinal ischemia/reperfusion injury [40] and a rat model of chronic ocular hypertension [42]). Thus, if naloxone acted through the opioid receptors it should have promoted (rather than ameliorated) neurodegeneration. Action via the opioid receptors may in fact explain why naloxone does not provide complete protection in our experiments.

Taken together, our results indicate that peripheral (non-eye related) bacterial activity and/or products are potential contributing factors to glaucoma pathophysiology through upregulation of the TLR4 and the complement system pathways and microglial activation in the affected tissues. The resulting optic nerve axon and RGC loss can be ameliorated by TLR4 inhibition. In addition, we demonstrate that oral bacterial load in patients who have glaucoma is significantly higher than that of subjects without the disease. This suggests that patients with glaucoma may be exposed to higher levels of bacterial products which over time can potentially be a factor exacerbating the severity and/or progression of the disease.

Since acceleration of CNS neuronal loss due to infectious/inflammatory processes in the periphery is not specific to glaucoma [9]–[11], commensal microbial flora may have similar effects in other neurodegenerative diseases. It is indeed intriguing that artificially-induced periodontitis, in an animal model of AD leads to exacerbation of the disease pathology [43]. Based on the studies cited together with the current work, we propose that chronic peripheral inflammation due to chronic exposure to microbial products caused by disturbances of the local microbiom in the oral cavity can lead to progression of neurodegeneration that has been initiated by genetic and other epigenetic or environmental factors (for example high IOP in the case of glaucoma). This secondary neurodegeneration can potentially progress in the absence of the factors that initiated CNS pathology and may account in part for the chronicity and periodic exacerbations that are typical in the course of neurodegenerative conditions. If proven, such a hypothesis can have significant therapeutic and public health implications.

## Materials and Methods

### Ethics Statement

All experiments involving human subjects were approved by the Institutional Review Board of SUNY Downstate Medical Center (IRB # is 266751-6). All animal experiments were approved by the Institutional Animal Care and Use Committee of SUNY Downstate Medical Center.

### Subcutaneous LPS administration

Mice were anesthetized using a mixture of isoflurane and oxygen. Five mg of LPS from *Salmonella typhimurium* (Enzo Life Sciences) was dissolved in 2 mL sterile distilled H_2_O. 30 µL of the LPS solution (60 µg LPS) was injected subcutaneously into a hind footpad of each experimental mouse. Mice were allowed to recover from anesthesia before being returned to their cages. Mice from the control group similarly received 30 µL of sterile 0.1M phosphate buffered saline (PBS). LPS administration was at 6 months of age for DBA/2J animals and at 11 weeks of age (one week prior to microbead induced IOP elevation) for C57BL/6 mice.

### Microbead-induced IOP elevation

Male C57BL/6 mice (12-weeks old) were subjected to uniocular microbead-induced IOP elevation as previously described [18] using 10 µm polysterene microbeads. IOP elevation was verified 3–7 days after microbead injection in the anterior chamber. All animals received a second injection of microbeads in the same eye 3 weeks after the initial injection.

### IOP measurement

Intraocular pressure was measured in mice noninvasively at least every two weeks using a rebound tonometer as previously described [44]. Briefly, the animals were held in a custom-made restrainer that does not compress the chest or neck while IOP is measured [45]. IOP measurements were performed after topical application of 0.5% propacaine. A series of five consecutive measurements per eye were obtained and averaged. Only measurements with the standard deviation of less than 10% were used. The tonometer readings were then converted to IOP values (expressed in mmHg) using a mathematical formula specific for the strain of mice and for the tonometer used.

### Naloxone administration

Animals received naloxone (0.13 mg/day) via osmotic minipumps inserted subcutaneously. 28-day minipumps (model 2004, Alzet, Cupertino, CA) were inserted under general isoflurane anesthesia 2 days prior to LPS administration. Minipumps were replaced 28 days later. RGCs were retrogradely labeled with fluorogold 56 days after LPS administration and animals were sacrificed 4 days later (62 days after minipump insertion).

### Immunohistochemistry

#### Cryosections

Tissue was fixed in 4% PFA in 0.1M PBS for 15 minutes at room temperature, cryopreserved in 10% sucrose overnight and 20% sucrose for 2 hours and blocked in cryomatrix for sectioning on a cryostat. Eight micron sections were obtained, rinsed with PBS and incubated in a blocking buffer (5% donkey serum, 1% BSA, 0.3% Triton-X in 0.1M PBS) for 1 hr at room temperature. The sections were then incubated with a primary antibody in an antibody buffer (1% donkey serum, 1% BSA, 0.3% Triton-X in 0.1M PBS) overnight at 4 C. Rabbit polyclonal anti-Iba1 antibody (WAKO, Richmond, VA; product code: #019-19741) was used at the dilution of 1∶1000 of the supplied concentration; rat monoclonal anti-CD11b antibody (Developmental Studies Hybridoma Bank, Iowa City, IA; product code: M1/70.15.11.5.2) was used at the dilution of 1∶5 of the supplied concentration. Sections were washed three times for 10 minutes with 0.1M PBS and incubated with an appropriate Alexa-Fluor conjugated secondary antibody for 2 hours at room temperature. After two five-minute washes in 0.1M PBS, incubation in a solution of 4′,6-diamidino-2-phenylindole (DAPI) for 5 minutes and two additional washes in PBS for 5 minutes, the sections were coverslipped using a glycerol mounting medium containing p-phenylenediamine (PPD) as an anti-fade agent.

#### Retina whole-mounts

Animals were perfused with 4% PFA, the eyes were enucleated and the retinas were dissected as whole-mounts. They were rinsed with PBS and incubated in a blocking buffer (5% donkey serum, 1% BSA, 0.3% Triton-X in 0.1M PBS) overnight at 4°C. Tissue was then incubated with primary antibody in an antibody buffer (1% donkey serum, 1% BSA, 0.3% Triton-X in 0.1M PBS) for 24 hours at 4°C. Rabbit anti-Iba1 antibody (same as above) and rat anti-CD68 antibody monoclonal antibody (AbD Serotec; MCA1957) were both used at the dilution of 1∶500 of the supplied concentration. Tissue was washed at least four times for 30 minutes with PBS and incubated with an appropriate Alexa-Fluor conjugated secondary antibody in the antibody buffer for 24 hours at 4°C. Finally, whole-mounts were washed twice in 0.1M PBS, incubated in DAPI solution for 20 minutes, washed again twice in PBS, placed on glass slides and coverslipped with the PPD containing mounting medium.

### Optic nerve microglial cell counts

Cryosections containing a longitudinal portion of the optic nerve that included the optic nerve head were imaged on an epi-fluorescent microscope (AxioImager Z1, Zeiss Thornwood NY) equipped with a digital camera (Axiocam HR, Zeiss Thornwood NY) using a 20× lens and proper excitation filter for the secondary antibody used. Stained cells were counted manually on collected images as follows: Images were imported into Adobe Photoshop with different Z-stack planes (four) of the same optic nerve section pasted as individual layers. An area corresponding to the anterior 200 µm of the optic nerve was outlined taking into account the microscope- and lens- specific scaling. Nuclei (DAPI stained) of Iba1-positive and CD11b-positive cells were counted in all available Z-plane images of the optic nerve section taking care not to count nuclei of the same cell in different Z-stack planes multiple times. Cell counts were entered into Excel (Microsoft, Redmond CA) for graphing. Statistical analysis was performed using the NCSS software package (NCSS, Kaysville UT).

### Microglial cell counts in retinal whole-mounts

16 fields per retina were acquired with a laser scanning confocal microscope (Olympus, FV1000) using 40× immersion-oil lens (numerical aperture of 1.3) and appropriate laser wavelength. Kalman filtering (with two count integration) was used for noise reduction. Images were acquired with the resolution of 0.31 µm/pixel as a 30 to 60 µm Z-stack with 2 µm slice thickness. Each quadrant was sampled with 4 fields located 500 µm, 1000 µm, 1500 µm, and 2000 µm away from the center of the retina. Total number of Iba1+ microglial and Iba1+ amoeboid microglial cells were counted manually in the four most proximal to the ONH fields of each quadrant per retina (n = 5 eyes for the control group; n = 7 eyes for the LPS-treated group); only cells that had their nuclei in NFL or RGC layers were counted.

Total number of Iba1+/CD68+ amoeboid microglial cells as well as CD68+-only amoeboid microglial were counted manually in all sixteen fields per retina (n = 4 eyes for the control group; n = 6 eyes for the LPS-treated group). Again only cells with nuclei in the NFL or RGC layers were counted.

### Morphometric analysis of microglial activation

The original multi-channel Z-stack images were analyzed in a masked fashion. To ensure consistency of cell sampling, all Iba1^+^ cells analyzed were selected using the following criteria: a) cells had to be located closest to the center of an image; b) cells had to be located in the NFL or in the innermost part of the RGC layer; c) only cells with at least 2 primary processes were analyzed (no amoeboid cells were included in the analysis). After the cell selection process, the images were saved as 8-bit grayscale tiff files and loaded as an image sequence into the open source Fiji software (http://fiji.sc/Fiji). Tracing of the microglial cells was manually performed in Fiji. The Simple Neurite Tracer plugin was used to manually trace all visible primary, secondary, tertiary, and so on, processes of each cell in the entire Z-stack taking particular care not to include in the trace closely opposed projections from the neighboring cells (as opposed to utilizing a maximum intensity projection image in which distinction between processes from adjacent cells is usually impossible). Traces then were saved as a separate file and converted to a binary image. Following that, the cell trace position was adjusted so that the center of each traced cell nucleus was placed exactly at the center of the image.

#### Sholl analysis

Sholl analysis of the resultant binary files was performed in Fiji software using Sholl Analysis tool with the following settings: Sholl method = Intersections, starting radius = 25 pixels (7.75 um), ending radius = 255 pixels (79.05 um), radius step size = 10 pixels (3.1 um), samples per radius = 1.

#### Skeleton Analysis

Traces of the same microglial cells used for Sholl analysis were also skeletonized in Fiji using the Skeleton Plugin. Cell skeletons were then analyzed using the “Analyze Skeleton” command.

#### Morphometric analysis

Analysis of cell solidity (area of the cell [*Ac*] divided by the area of the convex hull), convexity (perimeter of the convex hull divided by the perimeter of the cell [*Pc*]) form factor (defined as equal to *4*×π×*Ac*/*Pc^2^*), roundness, aspect ratio (defined as the ratio of the major to minor axis length”) were performed using measurements obtained in Adobe Photoshop 7.0 (AdobeSystems, San Jose CA) (using the FoveaPro plugin (Reindeer Graphics, Inc, Asheville, NC). To check the accuracy of the measurements by FoveaPro, several sample images were also analyzed in Fiji using the FracLac plugin (with the “Calculate Hull and Circle metrics” option enabled). Values of the measurements between FoveaPro and Fraclac differed less than 1.3%.

Results of the various microglial morphometric measurements were transferred to an Excel worksheet (Microsoft, Redmond CA) for graphing and further analysis.

### Quantitative Real-Time Polymerase Chain Reaction (qPCR)

Animals were perfused transcardially with ice-cold, sterile 0.1M phosphate buffer, pH 7.4 (PBS), and tissue was immediately collected. For retina collection, eyes were enucleated and retinas were dissected in ice-cold PBS. Immediately following tissue collection, tissue samples were placed into the RNA stabilizing agent - RNAlater (Life Technologies, Grand Island, NY) - and stored at −80°C until used or immediately processed using Trizol reagent (Life Technologies, Grand Island, NY) according to the manufacturer's instructions. Briefly, tissues were homogenized in the Trizol, chloroform was added to separate the proteins from the RNA. After centrifugation, the RNA-containing aqueous phase was aspirated, and the organic phase was used for protein extraction. The RNA was precipitated with isopropanol, washed in 70% ethanol, and column purified using a kit (RNAeasy kit; Qiagen, Valencia, CA).RNA concentration and purity was assessed using a NanoDrop N100 spectrophotometer (NanoDrop Technologies, Wilmington DE). Only samples with a 260/280 ratio above 1.7 were used for later assays. One microgram of total RNA from each sample was reverse transcribed to cDNA using random hexamer primers with a kit (Qiagen, Valencia, CA). Two µL of cDNA (1∶5 dilution of the original concentration) from each sample was used for real time polymerase chain reactions (RT- PCR) with Power SYBR Green master mix (Biobasic, Ontario, Canada) and specific primers for the genes under investigation (Table S1 in File S1). The samples were analyzed on the Applied Biosystems 7900HT Fast Real-Time PCR System (Life Technologies, Grand Island, NY). Statistical analysis was be performed using comparative delta-delta Ct method using levels of RPS11 (40S ribosomal protein S11) housekeeping gene as a reference.

### Assessment of optic nerve damage

Animals were perfused transcardially with 4% ice-cold paraformaldehyde (PFA) in 0.9% PBS. After enucleation, the cranium was opened, and the brain together with the optic nerves was removed and immersed in a mixture of 1.2% PFA/0.8% glutaraldehyde in 0.9% PBS for 24 hours at 4°C. The fixative was washed out and the ONs were osmicated in 2% osmium tetroxide overnight and then embedded in epoxy resin (LR White; Electron Microscopy Sciences, Hatfield PA). Semi-thin sections were cut with an ultramicrotome, stained with p-phenylenediamine and mounted.

#### Semi-quantitative pathologic grading of ONs

ONs were observed under brightfield with a 63× oil-immersion lens. Nerves were graded in masked fashion for the amount of optic nerve axon damage on a 5-point scale (5 = severe damage, 0 = no obvious damage) based on the number of surviving axons and the presence of degenerating axons as previously described [46]. Grading was performed by two independent observers who were masked to the group that the animals belonged to. Differences in scores of more than 1 point were adjudicated by consensus among the 2 observers.

#### Semi-automated optic nerve axon counts

Quantitative assessment of optic nerve axon damage was performed using the method described by Marina et al [47] as follows. Photomicrographs of the entire optic nerve were taken using 63× oil immersion lens in a mosaic mode and then merged to produce a single image file. Using Photoshop software (Adobe Systems, San Jose CA) the outline of the optic nerve was traced and the area of the nerve in pixels was calculated. Areas of homogeneous damage (AHD) were identified and outlined. From each AHD a 500×500 pixel area was selected and saved in a separate file. This file was then processed in ImageJ software using an algorithm which provides quantification optic nerve the number of surviving axons in the AHD image being analysed. The total number of axons in the optic nerve was calculated based on the area of each AHD in pixels and the number of axons estimated for each AHD using the following equation:
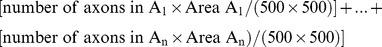



### RGC Labeling, Imaging, and Assessment of RGC loss

Mice were anesthetized by intraperitoneal injection of a mixture of xylazine/ketamine (10 and 5 mg/kg respectively). The skin over the cranium was incised and scalp exposed. Two holes (2 mm diameter) corresponding to the superior colliculi (SC) positions (4 mm posterior to the Bregma and 1 mm lateral to the midline [13]) were drilled with a Dremel tool. The SC were exposed by gentle aspiration of the overlying occipital cortex. Gelfoam soaked in 5% w/v aqueous Fluorogold (Fluorochrome, Denver) solution was directly applied to each SC. The gel foam was covered with antibiotic ointment, and the overlying skin sutured. After 4 days to allow uptake of the Fluorogold dye and retrograde transport to RGC somata, mice were euthanized. Eyes were enucleated and retinas surgically removed under a dissecting microscope. Retinas were prepared as flat whole-mounts on glass slides and fixed in 4% PFA for 15 minutes, then washed in balanced salt solution (BSS) and coverslipped using glycerol mounting medium containing p-phenylenediamine.

#### Semiquantitative RGC loss evaluation

Whole-mounted retinas were visually inspected under the microscope using 10× magnification. Retinae were graded based on the extent of RGC loss using a 10-point scale (10 corresponding to no obvious damage, 0 corresponding to complete RGC loss). Grading was performed by two independent observers who were masked to the group that the animals belonged to. Differences in scores of more than 2 points were adjudicated by consensus among the 2 observers.

#### Retinal Imaging and RGC counts

Retinal images were obtained using an epi-fluorescent microscope (AxioImager Z1, Zeiss Thornwood NY,) equipped with a digital camera (Axiocam HR, Zeiss Thornwood NY) using a wide band ultraviolet excitation filter (appropriate for Fluorogold). Eighty one non-overlapping, adjacent images covering the entire retina were taken at 10× magnification in a mosaic of 9×9 frames, and saved as a computer file for future analysis. Computer-aided counts were performed as previously described [13].

### Clinical Methods

Recruitment was performed after protocol approval from the SUNY Downstate Institutional Review Board as part of a study to investigate the role of environment on gene function in glaucoma. Signed informed consent was obtained from all subjects.

#### Subject recruitment and sample collection

Subjects with open angle glaucoma (n = 75) as well as a healthy control group (n = 67) were recruited from the SUNY Downstate Eye clinics. There were no specific age exclusion criteria for either group. Inclusion requirements for the glaucoma group were open angles, the presence of a characteristic glaucomatous visual field defect (e.g. arcuate defects or nasal steps respecting the horizontal midline or more advanced visual field loss (like central or temporal islands in the absence of other ocular pathology), and typical ONH cupping (cup to disc ratio (CDR)>0.8) in at least one eye based on clinical examination. All cases were either on IOP lowering agents or had in the past undergone glaucoma surgery. Subjects in the normal (control) group had no current or past IOP elevation, no significant ONH asymmetry and had CDRs<0.5 in both eyes.

Subjects were provided with 30cc of Scope ™ mouthwash solution. All mouthwash was collected in 15cc vials anonymized and frozen upon arrival to the lab (within a few hours). Vials were frozen at −80°C and maintained at that temperature until they were used for analysis of 16S RNA and genomic pyrosequencing of bacterial RNA.

The study population was more that 74% African Americans (AAs), ∼18% Hispanic and ∼4% White. Preliminary analysis revealed that bacterial loads in controls were different among different races. Since the vast majority of subjects were AAs (and numbers of subjects from other races could not be used for meaningful analysis) only data from AA subjects (45 Controls and 58 cases) were further analyzed.

#### Sample preparation and analysis

Total DNA was isolated using a bead beater as previously described [48] quantified using a Nanodrop ND-1000 Spectrophotometer, and frozen until analysis. Total bacteria levels (copy number) were quantified using a BioRad iCycler Real-Time Detection System, with an initial step of 98°C for 2 min, followed by 45 cycles of 98°C for 5 s and 64°C for 5 sec (fluorescent readings were taken at 64°C step), then a melt curve from 65°C to 95°C at increments of 0.5/5 s. 20 ul reactions were run in an optical grade 96 well plate, using 1× SsoFast EvaGreen Supermix (BioRad), 1 ul of undiluted template DNA, 0.4 uM each of primers, Eub338F (ACT CCT ACG GGA GGC AGC AG) and Eub518R (ATT ACC GCG GCT GCT GG), and RT-PCR grade water. Negative controls (1 ul of water instead of template DNA) were run for each plate, in duplicate. Standard curves were generated for each experiment as previously described [49], by simultaneously running reactions on serial dilutions of *P. gingivalis* genomic DNA containing 10^7^-10^1^ copy numbers of the 16srRNA gene, in triplicate. Standard curves were automatically generated by the iCycler software, by plotting threshold cycles (*C*_t_) vs. standards' copy numbers. The *C*_t_ of the samples was used to extrapolate their total bacterial copy number from the standard curves, as previously described [49]. Samples were measured twice and the copy numbers averaged. If the concentration of the sample fell outside the range of the standard curve, the reactions were repeated using 10-fold serial dilutions of the template. Bacterial load counts in cases were compared with those in controls after log transformation and normalization.

### Statistical analysis

Data from RT-PCR, cell and axon counts were subjected to either t-test when two groups were analyzed or to a one-way ANOVA followed by Tukey-Kramer post-hoc testing for more than two groups to determine whether there was a statistically significant difference between groups. IOP values were compared using repeated measurement ANOVA (RM-ANOVA). Changes in mRNA levels were expressed relative to control mRNA levels as fold changes ± SEM. The NCSS software package (NCSS, Kaysville UT) was used to perform these statistical analyses. Graphs were generated in Excel software (Microsoft, Redmond CA).

#### Linear discriminant analysis

Linear discriminant analysis (LDA) was performed in NCSS (using stepwise variable selection). The analyzed data consisted of the amounts of DNA from 86 bacterial families (normalized by the amount of total DNA in the sample) from the oral washouts from glaucoma and control samples. The prior probabilities used for LDA were 0.93 and 0.7 for controls and glaucoma cases respectively, based on approximate glaucoma prevalence in the population studied.

## Supporting Information

File S1 **This file contains Figure S1–Figure S6 and Table S1.** Figure S1. Semi-quantitative assessment of RGC and ON axon loss after peripheral LPS administration. Figure S2. The degree of peripheral inflammatory response after LPS administration correlates inversely with RGC survival. Figure S3. Influence of TLR pathway gene upregulation on ON axon damage in LPS treated animals. Figure S4. Figure S5. Microglial parameters assessed by skeleton analysis (a–d), morphometry (e–g), and Sholl analysis (h–j). Figure S6. qPCR analysis of microglial activation markers and markers of inflammation. Table S1. List of primers used for qPCR.
(DOC)
