# Supplementary material for: Oral Microbiome Link to Neurodegeneration in Glaucoma
Source: PLoS One. 2014 Sep 2;9(9):e104416. doi: 10.1371/journal.pone.0104416 (PMC4152129; doi:10.1371/journal.pone.0104416)
Supplement: File S1 — This file contains Figure S1–Figure S6 and Table S1. Figure S1. Semi-quantitative assessment of RGC and ON axon loss after peripheral LPS administration. Figure S2. The degree of peripheral inflammatory response after LPS administration correlates inversely with RGC survival. Figure S3. Influence of TLR pathway gene upregulation on ON axon damage in LPS treated animals. Figure S4. Figure S5. Microglial parameters assessed by skeleton analysis (a–d), morphometry (e–g), and Sholl analysis (h–j). Figure S6. qPCR analysis of microglial activation markers and markers of inflammation. Table S1. List of primers used for qPCR. (DOC) [file pone.0104416.s001.doc]

**Astafurov et al.**

Supporting Material

**Supplemental Figures and Tables**


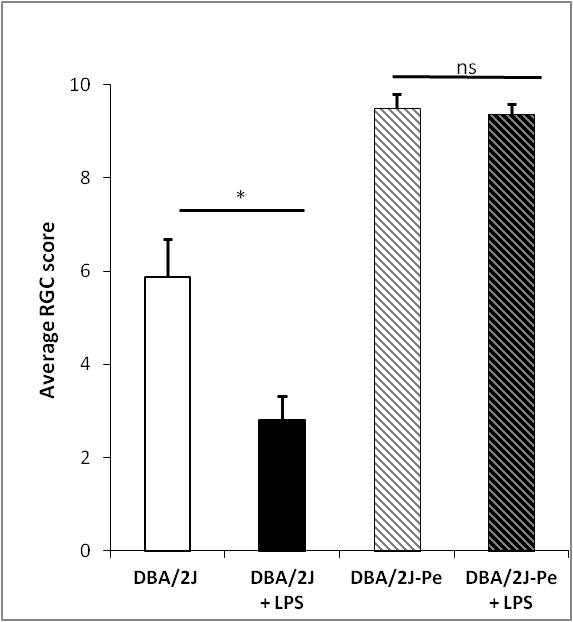

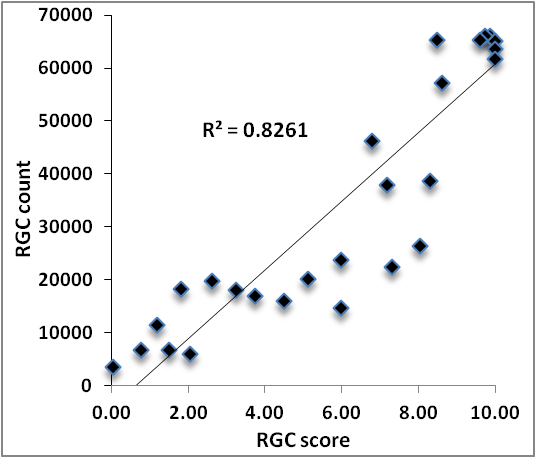

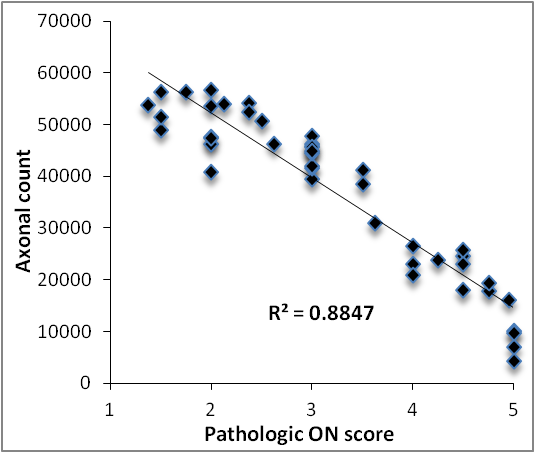

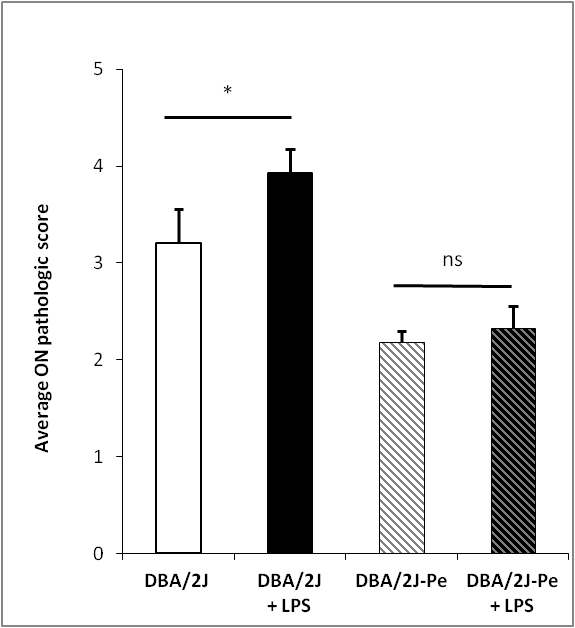


**d**

**c**

**b**

**a**

**Supplementary Figure S1.** Semi-quantitative assessment of RGC and ON axon loss after peripheral LPS administration. Six-month old male DBA/2J and DBA/2J-Pe mice were given a single subcutaneous injection of 60µg LPS (or PBS) into the hind footpad. Experimental and control animals (age-, sex- and strain-matched) were euthanized at 8 month of age after retrogradely labeling RGCs with fluorogold. Retinas were flat-mounted for RGC counting and ONs processed for ON axon counting.

**(a)** semi-quantitative scoring of the retinas by masked observers using a 10-point scale for the number of remaining RGCs. **(b)** ON damage assessment by semi-quantitative pathologic scoring of ON axon degeneration by masked observers on a 5-point scale. **(c)** There is good correlation (R^2^=0.83) between total number of RGCs and the semi-quantitative pathologic scores given by observers. **(d)** Similarly there is good correlation (R^2^=0.88) between total ON axon counts and the semi-quantitative score of ON damage given by observers.


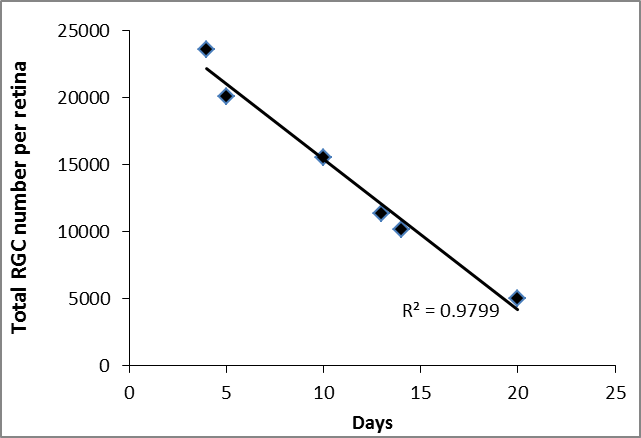


**Supplementary Figure S2.** The degree of peripheral inflammatory response after LPS administration correlates inversely with RGC survival. Six-month old male DBA/2J mice were given a single subcutaneous injection of 60µg LPS into one hind footpad. All animals exhibited variable degrees of transient weight loss. The number of days to regain pre-LPS administration body weight was correlated to the average number of surviving RGCs per animal at 8 months of age (n=6 animals).

**
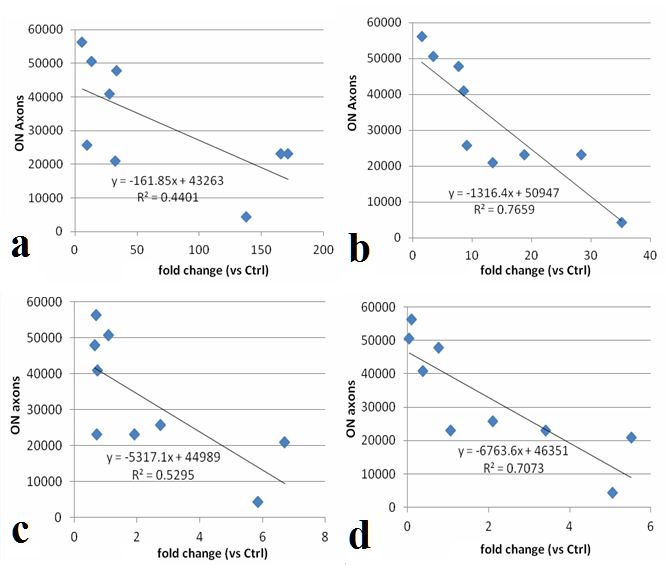
**

**Supplementary Figure S3.** Influence of TLR pathway gene upregulation on ON axon damage in LPS treated animals. Inverse correlation between the normalized amount of **a)** TLR4, **b)** Myd88, **c)** IRF3, **d)** TRIF mRNA (expressed as fold increase in LPS-treated versus control eyes) and the total number of ON axons. Squared correlation coefficients (R^2^) range from 0.44 to 0.766.


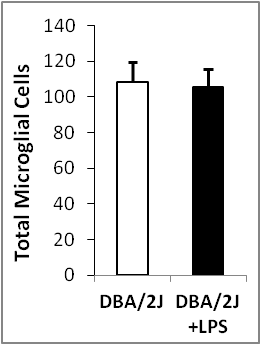

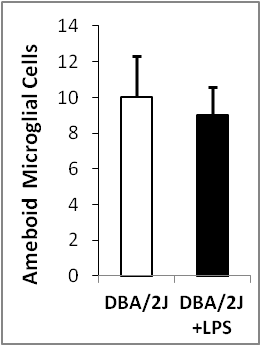

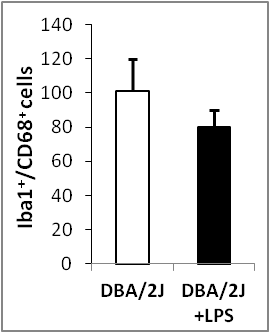

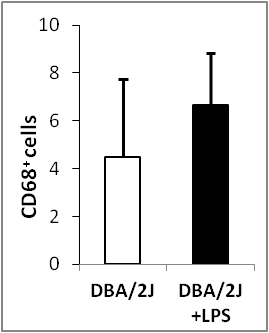


**a**

**b**

**c**

**d**

**e**

**f**

**g**

**Supplementary Figure S4.** Representative **(a)**anti-Iba1 **(b)**anti-CD68 and **(c)**merged images of microglial cells from the inner retina of LPS-treated DBA/2J mice. Numbers of **(d)** total Iba1+, **(e)** amoeboid Iba1+, **(f)** Iba1+/CD68+ and **(g)** CD68+ microglial cells counted per 16 sampled areas in all 4 retinal quadrants. All data are presented as mean + SEM. Differences between groups were not statistically significant (p > 0.05).


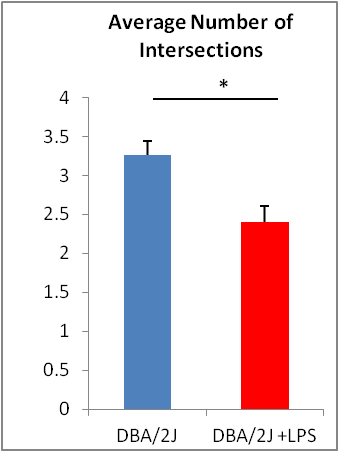

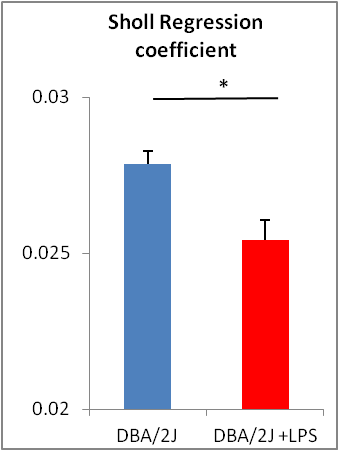

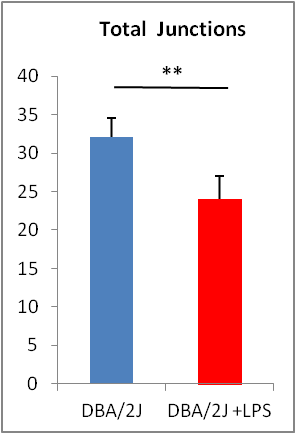

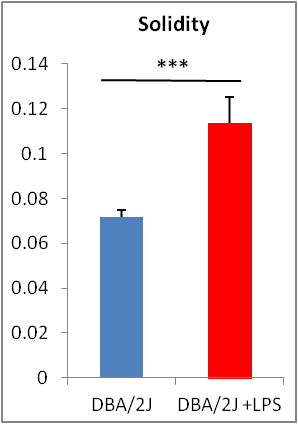

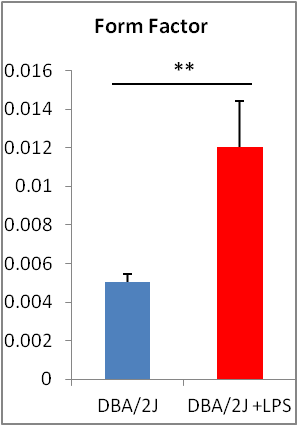

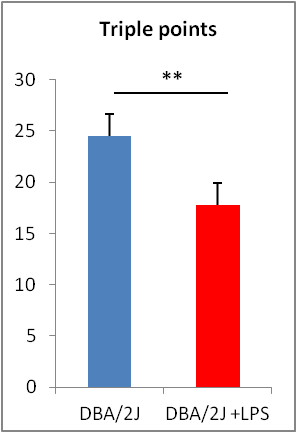

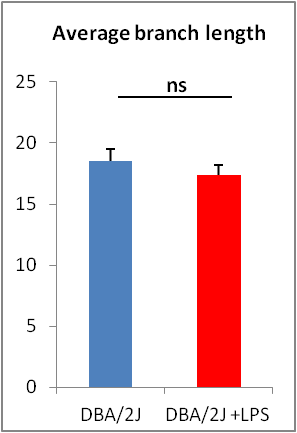


**a**

**h**

**b**

**c**

**d**

**f**

**g**

**i**

**j**

**e**

**Supplementary Figure S5.** Microglial parameters assessed by skeleton analysis **(a-d),** morphometry **(e-g),** and Sholl analysis **(h-j).** All data are presented as mean + SEM. Differences between groups were assessed using Mann-Whitney U test as many of these parameters do not follow normal distribution (*p < 0.05, **p < 0.01, ***p<0.001, ns > 0.05).


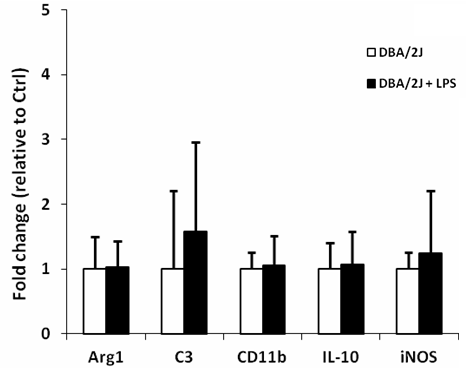


**Supplementary Figure S6.** qPCR analysis of microglial activation markers and markers of inflammation. Data were tested for statistical significance using t-test. No differences were detected (p>0.05) for all genes studied.

**Table S1. List of primers used for qPCR.**

| **Gene name** | **Forward (5'-3')** | **Reverse (5'-3')** |
| --- | --- | --- |
| ***RPS11*** | CGTGACGAAGATGAAGATGC | GCACATTGAATCGCACAGTC |
| ***C1qb*** | CCAACGCGAACGAGAACTAT | GTGGTCACCTGGAAGGTGTT |
| ***C3*** | ATAAAGAGCCAGCGGCTACA | GCCGTAGGACATTGGGAGTA |
| ***TLR4*** | AGGAGTGCCCCGCTTTCACC | GCCAGAGCGGCTGCTCAGAAA |
| ***Myd88*** | GCGAGCGTACTGGACGGCAC | TCCTCCGCCAGCAAGGTCCA |
| ***Traf6*** | CAGCGACCCACAATCCCACGG | CCAGGCATGGAAGGCACGGT |
| ***Trif*** | CTCGGGGTACCTGACGGGGC | TTCCAGCGTACCCGCTGTGC |
| ***Irf3*** | GGGTTGCGTGGCTGCGAGTC | CGTTCGGCTCTCGTCCAGCC |
| ***p38*** | GGATTTTGGGCTGGCTCGGCA | CAGCCCCTGGGGTTCCAACG |
| ***Junk*** | TGGCCAGTTTCTGCCGCACAC | GGGTGAGAGACGGTGCTGGGT |
| ***cJun*** | TACCAGTTCGCACAGGCGGC | TCCTTGGAGCCCGCAGACCA |
| ***IL-10*** | GGCTGAGGCGCTGTCATCGATTT | TGGCCTTGTAGACACCTTGGTCTTG |
| ***CD11b*** | CCCCACACTAGCATCAAGGG | GAGGCAAGGGACACACTGAC |
| ***Arg1*** | TGTGAAGAACCCACGGTCTG | ATGTCAGTGTGAGCATCCAC |
| ***iNos*** | CCTGCTTTGTGCGAAGTGTC | CTCTTGCGGACCATCTCCTG |
